# Peri‐operative pain management in major lower extremity amputation in vascular Surgery: a UK anaesthetic and vascular surgery Delphi consensus study*

**DOI:** 10.1111/anae.70107

**Published:** 2025-12-17

**Authors:** Thanapon Ekkunagul, Caitlin Sara MacLeod, Anna Celnik, John Chalmers, Ross Thomson, Alan J. R. Macfarlane, David Bosanquet, John Nagy, Patrice Forget, the Delphi expert panel, Abhijoy Chakladar, Ahmed Abidia, Alan Fayaz, Alan Macfarlane, Alasdair Taylor, Alastair Thomson, Alexander Bell, Amy Sadler, Andrea Harvey, Andrew Mitchell, Andrew Tambyraja, Andrew William Garnham, Andrey Varvinskiy, Ankur Thapar, Anna Celnik, Caroline Curry, Catherine Western, Catriona Semple, Christopher G Davies, Craig Forrest, David Bosanquet, Denis Harkin, Diane Rosemary Hildebrand, Douglas James MacKenzie, Emma Elizabeth Florence Scott, Emma Jannine Baird, Ferdinand Serracino‐Inglott, Fiona Myint, Frances Kent, Francesco Torella, Gareth Harrison, George A. Antoniou, George Edward Smith, Graeme A McLeod, Ian Chetter, Indran Raju, John Chalmers, John Nagy, John O'Donoghue, Jonathan Paul Seeley, Kaji Sritharan, Keith Hussey, Keith Jones, Kersten Morgan Bates, Kevin Graham Mercer, Kieran Murphy, Lisa Anne Grimes, Matt Metcalfe, Maureen Sweeney, Michael McCusker, Michael Neil, Michelle Lamont, Mohamed Elsherif, Nat Haslam, Naveeta Maini, Nicholas Stuart Greaves, Patrick Coughlin, Patrice Forget, Peter Merjavy, Robert Hinchliffe, Rob Sayers, Ross Thomson, Russell William Jamieson, Sachin R Kulkarni, Sandeep Bahia, Sandip Nandhra, Serena Goon, Tanim Siddiqui, Tim Stansfield, Vanessa Fludder, Vikas Kaura, Vishal Gupta

**Affiliations:** ^1^ Epidemiology Group, Institute of Applied Health Sciences University of Aberdeen Aberdeen UK; ^2^ Department of Vascular Surgery, Ninewells Hospital and Medical School Dundee UK; ^3^ Division of Cardiovascular and Diabetes Medicine University of Dundee Dundee UK; ^4^ Department of Anaesthesia, Aberdeen Royal Infirmary Aberdeen UK; ^5^ Department of Anaesthesia, Glasgow Royal Infirmary Glasgow UK; ^6^ School of Medicine University of Glasgow Glasgow UK; ^7^ South East Wales Vascular Network, University Hospital of Wales Cardiff UK; ^8^ Division of Population Medicine, School of Medicine Cardiff University Cardiff UK; ^9^ IMAGINE UR UM 103, Montpellier University, Anesthesia Critical Care, Emergency and Pain Medicine Division, University Hospital Centre Nimes Nimes France

**Keywords:** amputation, surgical, pain management, vascular surgery

## Abstract

**Introduction:**

Major lower extremity amputations occurring secondary to vascular disease remain prevalent worldwide. Pain surrounding these procedures is complex, multifactorial and associated with poor functional and psychosocial outcomes. The evidence base informing pain management approaches in major lower extremity amputations remain largely heterogeneous and limited. This study aimed to establish procedure‐specific, multispeciality consensus on the ideal principles and practices required to optimise pain management for vascular surgical patients undergoing major lower extremity amputations.

**Methods:**

A three‐round online modified Delphi consensus process was undertaken, with consultant anaesthetists and consultant vascular surgeons across the UK forming the expert panel. Structured statements were assessed on a 5‐point Likert scale against a strong consensus threshold of ≥ 75% ratings in agreement or disagreement, and a rating stability criterion of < 10% change between rounds. Free‐text responses were thematically analysed at each round to iteratively modify or generate new statements.

**Results:**

Seventy‐two panellists participated in the study. Of the 44 consensus statements assessed, 32 reached strong consensus agreement. These included: shared cross‐speciality responsibility for pain management; the mainstay role of locoregional analgesia; use of perineural catheters; opioid‐sparing approaches; and protocolised decision aids with individualisation of analgesia. Barriers to practices identified included resource constraints and the paucity of direct evidence. There was non‐consensus in 12 statements, notably on pre‐amputation initiation of locoregional analgesia; ultrasound‐guided nerve catheter placement; and surgeon‐delivered regional analgesia. No statement reached strong consensus disagreement.

**Discussion:**

This study provides the first procedure‐specific consensus, delineating agreed principles and preferred pharmacological and locoregional analgesic approaches to peri‐operative pain management in patients undergoing major lower extremity amputations. The areas of non‐consensus expose key uncertainties that may inform future research, service organisation and guideline development agendas.

## Plain Language Summary

**What we did:**

Groups of expert doctors from across the UK worked together online to agree on the best ways to manage pain for patients who need to have their leg removed. They answered questions over three rounds and rated how much they agreed with different statements. The goal was to see which ideas most experts supported.

**Why we did it:**

Some people need to have part of their leg removed because of problems with their blood vessels. These operations can cause different kinds of pain, which can make it harder for patients to move, heal and recover emotionally. Doctors use many methods to help with pain, but the research about the best ways to do this is mixed and not always clear.

**What we found:**

Seventy‐two doctors took part. They looked at 44 statements and strongly agreed on 32 of them. They agreed that doctors from different specialities should share responsibility for pain care, that numbing certain nerves is very helpful, that small tubes can be placed near nerves to give pain medicine and that treatment should try to reduce the need for strong opioid drugs. They also agreed that pain plans should be guided by clear rules but still be flexible for each patient. Some things did not reach agreement, such as starting nerve‐numbing treatments before surgery and which doctors should place nerve catheters. This study is the first to create clear, shared guidance on how to manage pain for people having major leg amputations. The areas where the experts did not agree show where more research and clearer rules are still needed.

## Introduction

Major lower extremity amputations are debilitating, life‐altering surgical interventions, most commonly performed as a consequence of peripheral arterial disease and/or diabetes in patients with significant multimorbidity [1, 2]. Outcomes following major lower extremity amputations are often poor, with significant functional and psychosocial debilitation, and 1‐year mortality rates can be as high as 48% [2, 3]. Despite an evolving understanding of the biopsychosocial determinants of limb loss and a growing emphasis on risk reduction strategies in vascular surgery, major lower extremity amputations remain prevalent globally with inconsistent trajectories of incidence observed between countries [4, 5].

For patients undergoing a major lower extremity amputation, pain is a defining and frequently preceding feature, driven by a convergence of ischaemic, nociceptive and neuropathic pathophysiology [6]. It influences the post‐major lower extremity amputation recovery course, where worse pre‐operative or acute postoperative pain is associated with poorer post‐amputation quality of life and increased risk of chronic pain syndromes [3, 7]. Pain management in patients undergoing a major lower extremity amputation remains suboptimal in routine practice. For instance, in the UK, where at least 4000 major lower extremity amputations are performed annually by vascular surgeons, the National Confidential Enquiry into Patient Outcome and Death (NCEPOD) report revealed that only one‐third of patients undergoing a major lower extremity amputation experienced ‘good’ postoperative pain control [8, 9]. A stakeholder consensus also identified effective pain management as a key practice and research priority for patients undergoing a major lower extremity amputation [10].

Whilst current guidelines support multimodal analgesic approaches to pain management in patients undergoing a major lower extremity amputation, a recent systematic review highlighted considerable heterogeneity in the available evidence supporting any particular modality [11]. Despite the availability of multiple options, including non‐opioid analgesics, pre‐operative gabapentin loading, epidural analgesia and peripheral nerve blockade (single‐shot or continuous catheters), pain management practices for patients undergoing major lower extremity amputations remain inconsistent [6, 11, 12]. This study aims to capture procedure‐specific anaesthetic and surgical insights and establish consensus on the ideal processes, needs and approaches to optimise pain management for vascular surgical patients undergoing major lower extremity amputations.

## Methods

**Figure 1:**
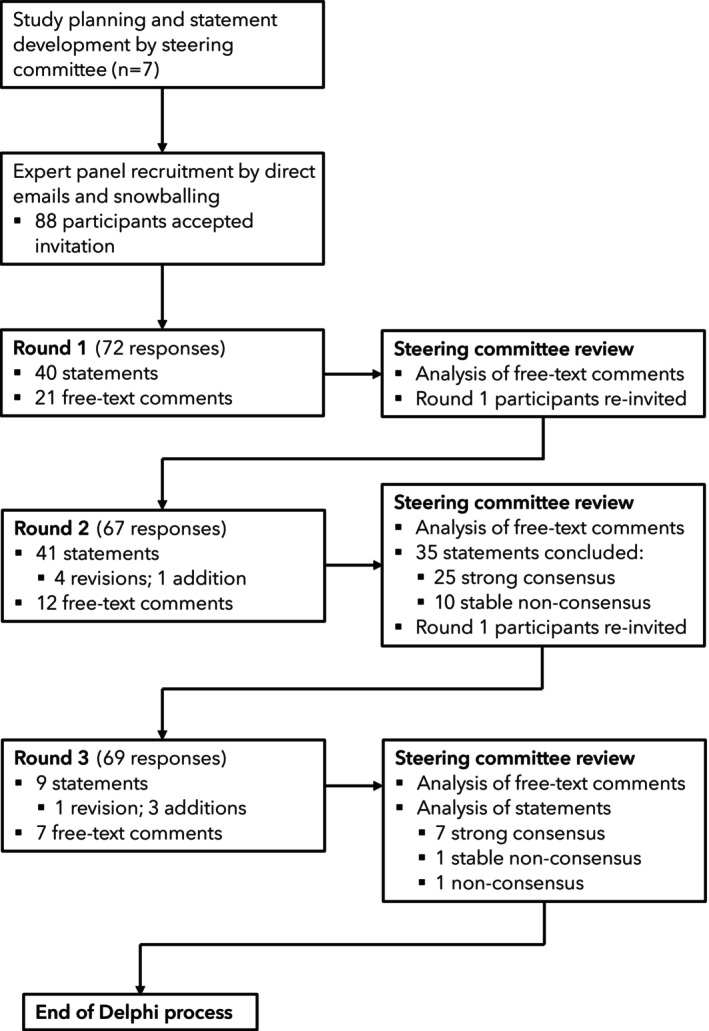
Study flowchart.

An online, modified Delphi consensus study was conducted in June and July 2024 and followed a previously published study protocol; no ethical approval was required [13]. The study was conducted in the UK, where healthcare is delivered through a publicly funded national health service model across all devolved nations. This setting minimises potential confounding heterogeneities that may arise from differing international health service structures whilst enabling a diversity of expert perspectives. A steering committee (TE, CSM, AC, JC, RT, JN, PF) oversaw the study process which comprised three iterative fortnightly rounds of structured, anonymised electronic surveys delivered via the Research Electronic Data Capture (REDCap; Vanderbilt University, Nashville, TN, USA) platform (Fig. 1) [14]. This study was conducted and reported in accordance with the CREDES guidance [15].

**Figure 2:**
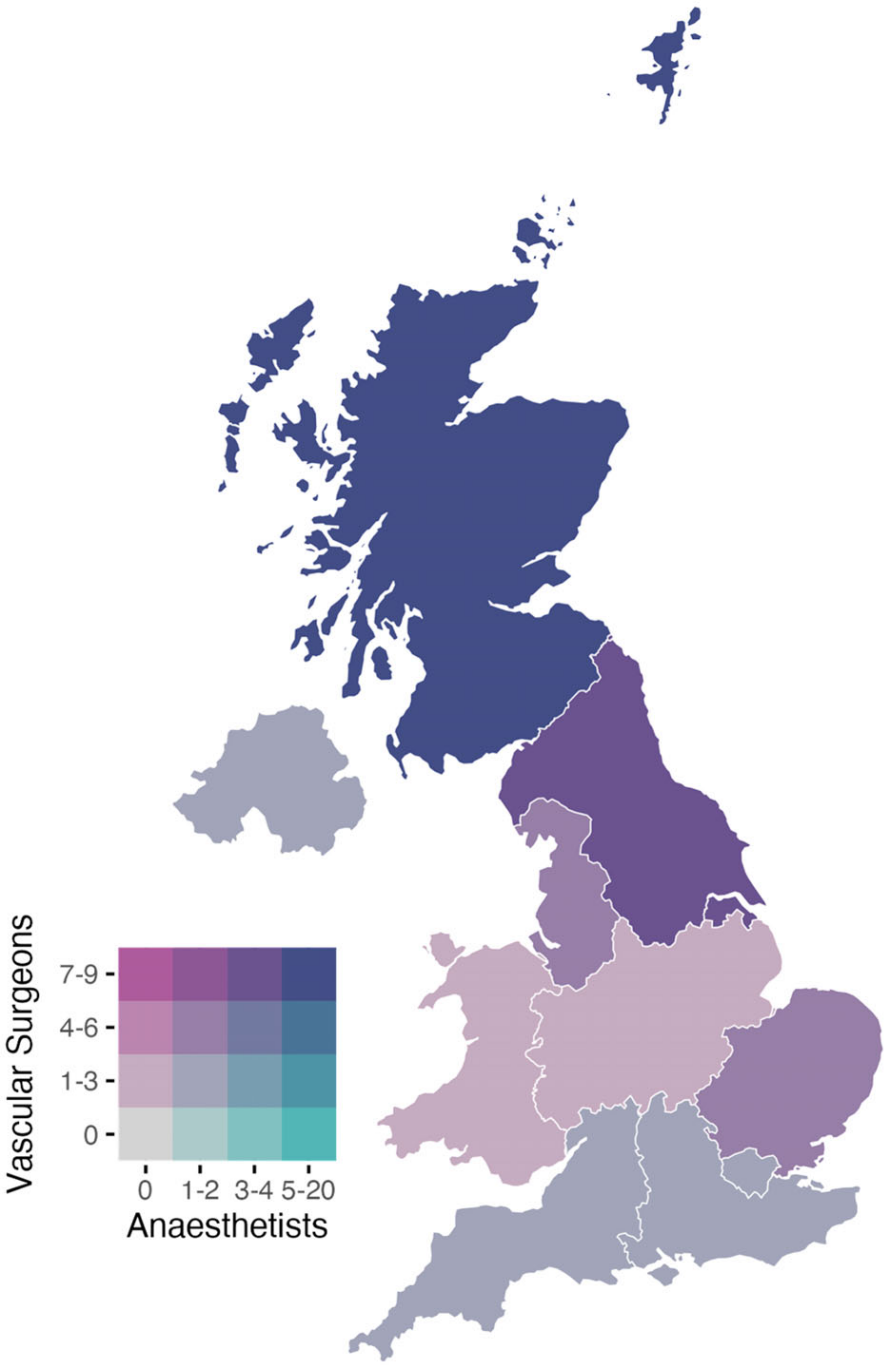
Geographical distribution of expert panellists (n = 72) across the 10 UK health regions: Scotland; Northern Ireland; North East and Yorkshire; North West; Midlands; Wales; East of England; London; South East; and South West.

The expert panel was defined as consultant vascular surgeons and consultant anaesthetists with specialist interests in vascular anaesthesia, regional anaesthesia or pain medicine (i.e. independent, unsupervised, final clinical decision makers in the respective specialities) who practice in the UK [13]. This composition of expertise reflects recommendations on best‐practice multidisciplinary peri‐operative management of patients undergoing major lower extremity amputations from the Vascular Society of Great Britain and Ireland, and the emphasis from existing guidelines on consultant‐level clinician involvement in this context [1, 16]. The panellist recruitment strategy has been described previously and involved targeted invitations via known networks of consultant clinicians and clinical academics, and the boards or registries of relevant national societies, with the use of snowball sampling [13]. This resulted in 72 consultant‐level clinicians participating in the study (Fig. 2, Table 1).

**Table 1:** Specialties and experience of the expert panel.

**Primary specialty**	**n = 72**
Vascular surgery	40
Anaesthetics	32
**Anaesthetic subspecialist areas**	**n = 32**
Vascular	20
Regional	19
Pain medicine	11
Major trauma	2
Intensive care	1
Peri‐operative medicine	1
**Years of experience since completion of training; y**	**n = 72**
< 5	20
5–10	17
10–15	13
15–20	8
≥ 20	14

Consensus statements were developed by the steering committee following an online literature search consisting of the keywords: ‘analgesia’; ‘pain management’; and ‘amputation’. Review articles, primary research articles and local, national or international pain management guidelines that discussed peri‐operative pain management approaches for patients undergoing major lower extremity amputations were identified and reviewed to inform statement generation. These were presented at the first Delphi round under three domains: perceptions and processes (exploring clinical practice priorities, ongoing research and practice needs, and speciality roles in major lower extremity amputation pain management); locoregional and intra‐operative techniques; and aspects of pharmacological pain management. Panellists rated their agreement with each statement using a 5‐point Likert scale. Free‐text comment fields were included to facilitate feedback. To accommodate the multispeciality composition of the panel, participants were given the option to abstain from statements focused primarily on surgical or anaesthetic techniques in the locoregional and pharmacological sections.

Following each round, aggregated responses and free‐text feedback were reviewed and analysed thematically by the steering committee to guide statement revision or generate new statements. Revised statements were presented in subsequent rounds with highlighted modifications. Summary distributions of the previous responses from the panel alongside previous ratings from individual participants were included with each statement for iterative feedback. The study was concluded after round 3. A final prioritisation round was not pursued due to the breadth of topics presented.

Aggregation of Likert score percentages was performed for each statement. A strong consensus criterion was defined a priori as ≥ 75% of panellists providing a 5‐point Likert scale rating in agreement (Likert 4 or 5) or in disagreement (Likert 1 or 2) with each statement. Between‐round changes in ratings were calculated to determine rating stability, based on an a priori criterion of < 10% absolute change in cumulative ratings in agreement and disagreement with each statement [17]. Both criteria were used to guide statement and study conclusion.

## Results

A total of 44 Delphi statements were assessed across three iterative rounds. Participation reached a maximum of 72 panellists in round 1 and 69 in round 3 (Fig. 1). Thirty‐two statements achieved strong final consensus agreement (Tables 2, 3, 4), with all but two of these statements (1.7, Table 2 and 3.4.1, Table 4) showing strong (< 10%) rating stability between rounds 2 and 3. Rating stability was not assessed for one statement (1.16, Table 2) as it was introduced in round 3. Rating distributions and participation levels by round are shown in online Supporting Information Appendix S2.

**Table 2:** Final agreement levels and rating stabilities of statements presented in the general principles, processes and approaches domain that achieved strong consensus agreement. Values are number, proportion.

	Final agreement	Change
1.1 Elucidating the best practices for pharmacologically managing pain in dysvascular major lower extremity amputation patients (i.e. pre‐, peri‐ and post‐amputation pain, including acute and chronic phantom limb pain) remains to be a key clinical practice challenge and research priority.	66/67, 99%	+1.3%
1.2 Existing international, national or local guidance relating to multimodal analgesic practices are not adequately comprehensive or specific to major lower extremity amputation pain management.	57/67, 85%	+5.9%
1.3 Multimodal pain management practices in major lower extremity amputation vary and are not fully guided by direct evidence.	62/67, 93%	+2.3%
1.4 More qualitative research on patient experiences and attitudes towards multimodal peri‐operative amputation pain management will help inform on analgesic strategies.	64/67, 96%	+2.5%
1.5 Peri‐operative multimodal analgesic management in major lower extremity amputation is a shared responsibility between the anaesthetics, pain and surgical teams.	66/67, 99%	‐1.5%
1.7 The approaches to major lower extremity amputation pain management should be personalised by considering biopsychosocial patient factors.	60/67, 90%	+10.4%
1.8 Analgesic initiation and escalation in post‐amputation pain should be titrated according to acute functional performance and biopsychosocial outcomes in addition to pain scores.	62/67, 93%	+9.2%
1.9 Pre‐operative pain management in patients undergoing major lower extremity amputation affects post‐amputation pain outcomes and should be as equally prioritised in routine clinical practice as post‐amputation pain.	67/67, 100%	+2.8%
1.10 Collaborative protocols involving the pain and surgical teams should be developed to facilitate early identification of patients due for major lower extremity amputation to initiate early pre‐operative analgesic review and optimisation.	66/67, 99%	+4.1%
1.11 Those who do not identify themselves as pain specialists but are involved in the clinical management of lower extremity amputation patients should be equipped with competencies to initiate, titrate and recognise the complications of certain non‐opioid multimodal analgesic drugs.	65/67, 97%	+5.3%
1.12 Decision aids for the peri‐operative management of pain should be made accessible for non‐pain specialists.	64/67, 96%	+1.1%
1.13 Peri‐operative pain management pathways for patients having major lower extremity amputation should be adapted locally to multispecialty departmental consensus and experience.	64/67, 96%	+5.2%
1.14 Patient education on post‐amputation pain symptoms, analgesic strategies and adverse effects should be incorporated into routine patient contact.	66/67, 99%	+2.7%
1.15 Where pain services are subdivided, acute pain specialists involved with post‐amputation pain management should be equipped with chronic pain management competencies for continuity.	58/67, 87%	+7.4%
1.16 The implementation of any potential peri‐operative pain management processes, approaches or modalities in major lower extremity amputation requires an appropriate dedication of resources at the local and regional levels.	65/69, 94%	N/A

Missing statement numbers are ones which did not achieve strong consensus.

**Table 3:** Final agreement levels and rating stabilities of statements presented in the locoregional and intra‐operative approaches domain that achieved strong consensus agreement. Values are number, proportion.

	Final agreement	Change
2.1 Locoregional analgesia should be the mainstay modality of pain management, where available, in major lower extremity amputations pre‐, intra‐ and postoperatively.	60/62, 97%	+8.7%
2.2 Continuous local anaesthetic infusion via a perineural nerve catheter is the preferred locoregional analgesia modality over single nerve blocks.	54/60, 90%	+0.4%
2.6 Continuous local anaesthetic infusion by perineural nerve catheters should be continued for an extended acute postoperative duration (e.g. > 72 h).	51/61, 84%	+6.3%
2.7 In above‐knee amputations, continuous local anaesthetic infusions should routinely target both the femoral and sciatic nerves.	50/60, 83%	+7.1%
2.8 In below‐knee amputations, the perineural nerve catheter should target the sciatic nerve proximally, via ultrasound guidance, at or above the popliteal fossa.	46/61, 75%	+0.4%
2.9 In below‐knee amputations, continuous perineural tibial nerve catheters placed intra‐operatively under direct vision may not provide complete anatomical coverage but may be preferable for practical reasons.	48/59, 81%	+4.8%
2.10 Where there may be contraindications to perineural nerve catheter placement, neuraxial (e.g. epidural) or alternative peripheral locoregional techniques such as single nerve blocks should be employed routinely.	57/61, 93%	+7.3%
2.11 Intra‐operative nerve transection techniques in tractional neurectomy vary by preference, but the body of evidence to support their effect on acute and chronic pain outcomes are scarce and equivocal.	35/39, 90%	+6.8%
2.12 The benefit of intra‐operatively incorporating alternative nerve‐handling techniques: targeted muscle reinnervation; regenerative peripheral nerve interface; or targeted nerve implantation, on pain outcomes should be investigated for feasibility and ability to implement.	35/42, 83%	+4.3%

Missing statement numbers are ones which did not achieve strong consensus.

**Table 4:** Final agreement levels and rating stabilities of statements presented in the pharmacological approaches domain that achieved strong consensus agreement. Values are number, proportion.

	Final agreement	Change
3.1 The comorbidities and potential risks of polypharmacy in patients undergoing major lower extremity amputation are barriers to the initiation, titration and escalation of multimodal analgesic drugs.	50/59, 85%	+7.0%
3.2 Multimodal analgesia in patients undergoing major lower extremity amputation should aim to be opioid‐sparing as much as possible.	55/59, 93%	+5.7%
3.3 The choice of pharmacological analgesic drugs in major lower extremity amputation pain management should be personalised by considering biopsychosocial patient factors.	57/58, 98%	+3.0%
3.4.1 Immediate release opioids are recommended as a rescue medication measure in post‐amputation and phantom limb pain management.	53/59, 90%	+17.4%
3.4.2 Continuous infusion or slow‐release opioids are not recommended as the mainstay of post‐amputation and phantom limb pain management.	47/59, 80%	+7.2%
3.6 The choice of pharmacological analgesic strategy I employ or prefer depends on their potential impact on chronic and phantom limb pain outcomes.	49/57, 86%	+8.5%
3.7 Non‐steroidal anti‐inflammatory drugs are efficacious and could be appropriately (meaning respecting the frequent absolute and relative contraindications) used more often in the management of acute nociceptive post‐amputation pain.	46/60, 77%	+2.1%
3.13 De‐escalation and weaning regimes for perineural catheters and all non‐opioid analgesics should be specific, standardised, and readily accessible.	55/59, 93%	‐0.5%

Missing statement numbers are ones which did not achieve strong consensus.

Twelve statements did not meet the threshold for strong consensus agreement or disagreement (Table 5). Of these, 10 were concluded in round 2 and one in round 3, as all agreement and disagreement ratings showed strong between‐round stability. Rating stability was not assessed for one statement (1.17) as it was introduced in the final Delphi round. Thematic summaries of free text comments are in online Supporting Information Appendix S3.

**Table 5:** Results and rating stabilities of statements that did not achieve strong consensus agreement or disagreement. Values are number, proportion.

	Final agreement	Change	Final disagreement	Change
**General principles, processes, and approaches**				
1.6 Peri‐operative pain management strategies in major lower extremity amputation patients should be guided by pain specialists.	43/67, 64%	+7.2%	6/67, 9%	‐9.1%
1.17 Where resources may be limited, the surgical team (i.e. surgical registrars and consultants) should also be equipped with competencies to deliver relevant locoregional analgesia (e.g. perineural catheters or nerve blocks).	48/69, 70%	N/A	13/69, 19%	N/A
**Locoregional and intra‐operative approaches**				
2.3 Perineural catheter placement under ultrasound guidance may provide greater analgesic reliability than intra‐operative placement under direct visualisation, where expertise is available.	17/59, 29%	‐0.9%	18/59, 31%	‐5.4%
2.4 Percutaneous perineural catheter placement under ultrasound guidance should be particularly indicated where there are concerns with amputation site infection.	41/61, 67%	+4.1%	7/61, 12%	‐8.5%
2.5 Continuous local anaesthetic infusion by perineural nerve catheters or other locoregional analgesia modalities should routinely be initiated when surgical indication for amputation is confirmed.	33/61, 54%	+1.1%	11/61, 18%	‐4.7%
2.13 Epidural analgesia should be considered as a locoregional analgesic modality in the ward setting pre‐ and postoperatively.	23/64, 36%	‐9.2%	30/64, 47%	+8.2%
**Pharmacological approaches**				
3.5 The potential adverse effects arising from utilising non‐opioid multimodal analgesic drugs are a greater concern to me than the adverse effects arising from opioids.	13/59, 22%	‐1.8%	25/59, 42%	+1.1%
3.8 Nefopam should always (if not contraindicated) be employed as an adjunctive analgesic drug for acute post‐amputation pain.	8/38, 21%	‐3.9%	15/38, 40%	‐5.5%
3.9 Pre‐operative initiation of (pre‐medication with) gabapentinoids should be routine unless there are concerns with increased sedation risk.	35/55, 64%	‐1.4%	7/55, 13%	‐3.9%
3.10 Oral alpha‐2 agonists (e.g. clonidine) should be routinely incorporated into the analgesic management regime as an escalation and rescue measure, particularly in patients who are non‐opioid naïve; opioid and opiate withdrawal; or patients with anxiety.	27/41, 66%	‐0.1%	5/41, 12%	‐1.4%
3.11 Oral N‐methyl‐D‐aspartate receptor antagonists (e.g. low dose oral ketamine) should be incorporated routinely into the multimodal analgesic management regime as an escalation and rescue measure, particularly where there are high risks of opioid tolerance or phantom limb pain.	27/42, 64%	+2.1%	4/42, 10%	‐3.8%
3.12 Parenteral calcitonin has a favourable safety profile and should be incorporated as an acute post‐amputation pain management measure.	6/25, 24%	‐6.8%	9/25, 36%	+1.4%

## Discussion

This study presents the first procedure‐specific consensus addressing the organisation and delivery of peri‐operative pain management in patients undergoing major lower extremity amputations in vascular surgery. These insights from a UK panel of anaesthetists and vascular surgeons highlight current challenges and emerging practices while identifying areas requiring attention to advance this understudied area of clinical practice.

There was strong consensus agreement that the paucity of existing evidence contributes to practice variability in pain management in patients undergoing a major lower extremity amputation (93%) and that adequate major lower extremity amputation‐specific guidance is lacking (85%). This limitation is not unique to major lower extremity amputations, as surgical pain management guidelines often pool evidence from differing surgical contexts and consequently may overlook procedure‐specific characteristics, prompting initiatives such as the European PROSPECT guidelines [18]. However, the challenge in generating evidence in this context may arise from the high multimorbidity burden of vascular surgical patients with consequential heterogeneity in pain management requirements and safety considerations [19]. This comorbidity burden was identified as a barrier to greater utilisation of multimodal analgesia among panellists (85%). Patients who are frail and have multiple morbidities are often characterised poorly or under‐represented in analgesic studies, limiting the applicability of existing evidence and the ability to synthesise analgesic safety data robustly to inform practices [20].

In addition to limitations in the current evidence base, there was strong agreement that resource allocation was a significant barrier to implementing optimal pain management strategies (94%). Geographical disparities in major lower extremity amputation rates have been observed consistently between and within countries; within the UK, analyses have shown that such discrepancies cannot be explained by patient‐level or socio‐economic factors [5, 21]. This may implicate systemic variations in healthcare provision and mirrors the inconsistencies in acute pain service models, staffing and training that have been reported in various countries [22]. Within this context, there was near‐unanimous agreement that pain management responsibilities should be shared between specialities (99%), complemented by education of non‐pain specialists (97%) and structured decision aids (96%). However, a network meta‐analysis of acute pain service models showed that services supported by pain specialist supervision are associated with greater improvements in postoperative pain outcomes [23]. Further work will be required to define collaborative models that expand competencies and clarify scopes of practice across specialities.

At the procedural level, there was 97% agreement on the mainstay role of locoregional analgesia in peri‐operative pain management of patients undergoing a major lower extremity amputation, with continuous perineural catheters being the preferred modality (90%). This contrasts with the variable uptake of perineural catheters in patients undergoing major lower extremity amputations reported in an international survey [12]. Nevertheless, systematic reviews have shown significant reductions in acute postoperative pain with the use of perineural catheters, albeit with low‐quality evidence and uncertainty over longer‐term pain outcomes [11, 24].

Panellist views diverged on the implementation of locoregional analgesia as there was no consensus on early, pre‐amputation initiation of locoregional analgesia (54%) or ultrasound‐guided perineural catheter placement (29%). Comparative evidence is scarce, with one small randomised study reporting no difference in acute postoperative pain reduction or phantom limb pain incidence between pre‐operative and surgically placed sciatic nerve catheters [25]. Limited operator expertise was also identified as a barrier to ultrasound‐guided catheter placement in this setting [12]. These findings reflect the broader challenges encountered in delivering regional anaesthesia services, including variabilities in training, implementation and resources [26]. Additionally, the non‐consensus on surgeon‐delivered locoregional analgesia (70%) underscores an ongoing debate between increasing patient access, patient safety and the dilution of anaesthetic training [27]. Further comparative evidence will be required to address these areas of non‐consensus.

Emerging perspectives were also observed, including the low panellist agreement on pre‐ and postoperative use of epidural analgesia (36%) and the strong interest on evaluating the role and impact of surgical techniques, namely nerve transection (90%) and nerve reinnervation (83%), on post‐amputation pain outcomes. Whilst evidence for intra‐operative nerve transection approaches is lacking, a meta‐analysis of current observational studies suggests that reinnervation techniques performed at the time of amputation may improve post‐amputation pain outcomes [28].

In the pharmacological domain, the consensus from the panel aligns with current opioid stewardship principles and a recent consensus from the USA favouring non‐opioid‐first analgesic strategies in vascular surgery [29]. Prolonged‐release opioid use was discouraged (80%), consistent with established international consensus [30]. However, despite a strong agreement on opioid‐sparing regimens (93%), consensus on specific non‐opioid analgesics was less clear. Cautious agreement was observed on expanded postoperative non‐steroidal anti‐inflammatory drug (NSAID) use (77%), highlighting ongoing uncertainty surrounding its safety in vascular surgical patient cohorts. A systematic review of analgesic‐related harms in patients with multimorbidity suggested that the certainty of harm estimates for NSAIDs is limited by the sparsity of randomised data and inconsistent characterisation of high‐risk patients [20]. Increased NSAID adoption would require careful, individualised stratification and more patient and procedure‐specific safety data.

No strong consensus was established on the routine use of gabapentinoids (64%); ketamine (64%); clonidine (66%); nefopam (21%); or parenteral calcitonin (24%). Most panellists agreed that multimorbidity and polypharmacy were barriers to utilising such drugs (85%), but there was no consensus on their perceived harm over opioids (22%). Limited familiarity and sparse major lower extremity amputation‐specific evidence may explain these conclusions. Whilst a recent network meta‐analysis of pre‐emptive analgesia identified gabapentinoids among the interventions associated with reduced postoperative pain and rescue analgesia in other surgical contexts, a pooled analysis of two small randomised controlled trials on gabapentin use in post‐amputation pain showed a modest effect size (number needed to treat = 8.9) and low certainty of clinically meaningful pain reduction [31, 32]. In addition, evidence for phantom limb pain reduction remains inconclusive [11].

Similarly, postoperative opioid dose reductions were observed in meta‐analyses of peri‐operative ketamine or clonidine use, but procedure‐specific data for acute post‐amputation pain are lacking [33, 34]. Opioid‐sparing effects have also been seen with nefopam use in other acute surgical settings, but with limited and uncertain efficacy; it is also absent in many pain guidelines due to international regulatory differences [35]. Finally, a systematic review of small randomised controlled trials suggested that calcitonin may provide short‐term relief of acute phantom limb pain, but inconsistent results and small sample sizes highlight a need for adequately powered studies [36]. Thus, although 86% of panellists agreed that analgesic choices should consider their anticipated impact on chronic and phantom limb pain outcomes, current pharmacological evidence remains insufficient to guide such decisions.

Beyond the modality‐specific findings, there was strong consensus agreement on individualised analgesic selection (98%) and titration to patient‐level biopsychosocial or functional characteristics (93%). These views align with the emerging promotion of holistic patient phenotyping to guide targeted analgesic strategies, particularly as pre‐operative psychological factors such as anxiety, depression and catastrophising predict worse postoperative pain outcomes [37]. The international IMI‐PainCare PROMPT consensus reinforces this approach by establishing a core outcome set of patient‐reported outcome measures for peri‐operative analgesic efficacy that incorporates physical, behavioural and affective components of the pain experience [38]. However, although psychological morbidity is a component of the current major lower extremity amputation core outcome set, a systematic review of amputation‐specific patient‐reported outcome measures found that available instruments remain limited and focused primarily on those who used prosthetics [39].

Qualitative studies emphasise the complexities for patients undergoing a major lower extremity amputation experience, where anxiety, information overload and fear of opioid adverse effects predominate patient perspectives on analgesic management [40]. However, patients also report reassurance when interventions such as perineural catheters are offered. Reflecting these complexities, there was strong consensus supporting pre‐operative analgesic assessment and counselling (99%), with unanimous agreement on the importance of pre‐operative pain management (100%). Panellists also strongly agreed on the need for more qualitative studies to guide major lower extremity amputation pain management strategies (96%). This may support the development of psychometric measures to inform assessment and analgesic selection within major lower extremity amputation pain management pathways.

There are limitations to this study. Whilst panellist expertise was standardised by the recruitment of consultant‐level clinicians, the results may not reflect the perspectives of the wider multidisciplinary team involved in the care of patients undergoing major lower extremity amputations. In addition, orthopaedic and plastic surgical perspectives were not included, limiting transferability of the results beyond vascular surgical pathways. Although panellist invitations through professional networks were disseminated without intended regional bias, snowball sampling may have introduced selection bias towards participants within established clinical or academic collaborations, limiting the diversity of perspectives and geographical representation. Nevertheless, snowballing was applied across all invitations to maximise the recruitment and representativeness of individuals who are regarded as possessing relevant expertise and interest in this clinical practice area. The uneven distribution of panellists by geography and subspecialty may limit the context sensitivity of the results to regional practices and service structures. However, the concentration of panellists in northern regions of the UK mirrors the north–south disparities in major lower extremity amputation rates observed in existing epidemiological studies and National Vascular Registry data [8, 21]. Representation from these regions with higher procedural volumes may alternatively support the practical relevance and clinical applicability of the consensus findings. Statement participation also varied where abstentions were permitted, but this was intended to preserve participation from those with specific interest and expertise. Finally, the Delphi methodology synthesises expert opinion and should be interpreted alongside, and not in place of, high‐quality evidence.

In conclusion, this Delphi consensus study provides a multispecialty perspective on the ideal approaches and current challenges to improving peri‐operative pain management for patients undergoing major lower extremity amputations in vascular surgery. Strong consensus was reached on: cross‐speciality responsibility; the mainstay role of locoregional analgesia; opioid‐sparing pharmacological approaches; the need to explore surgical techniques further; and a need for holistic patient assessment. The barriers and non‐consensus areas identified reveal gaps in the current comparative evidence base, resourcing and training. These findings may be translated into service development approaches to strengthen major lower extremity amputation‐specific peri‐operative pathways, formalising and normalising the role of each specialty in the initiation and maintenance of diverse multimodal analgesic approaches. The expansion of these pharmacological and locoregional analgesic capabilities would require a multidisciplinary approach, including incorporating these competencies into anaesthetic and surgical training curricula, ward staff education and ensuring integration with acute pain service structures. However, whilst multiple principles were agreed upon, the findings also highlight the increasing nuances of pain management in this context that will require further research and debate to inform clinical translation.

## Supporting information


**Appendix S1.** The Delphi expert panel.

**Appendix S2.** Delphi rounds 1–3 results breakdown.
**Appendix S3.** Thematic summary of panellist free text comments.
