# Supplementary material for: Peri‐operative pain management in major lower extremity amputation in vascular Surgery: a UK anaesthetic and vascular surgery Delphi consensus study*
Source: Anaesthesia. 2025 Dec 17;81(5):664–74. doi: 10.1111/anae.70107 (PMC13065886; doi:10.1111/anae.70107)
Supplement: Supplementary file 1 — Appendix S1. The Delphi expert panel. [file ANAE-81-664-s002.docx]

**Appendix S1:** The expert panel (study collaborators and participating steering committee or writing committee members).

1. Abhijoy Chakladar, University Hospitals Sussex NHS Foundation Trust, Brighton & Haywards Heath
2. Ahmed Abidia, The Princess Alexandra Hospital
3. Alan Fayaz, University College London Hospital NHS Foundation Trust/University College London
4. Alan MacFarlane, University of Glasgow
5. Alasdair Taylor, Ninewells Hospital and Medical School, Dundee
6. Alastair Thomson, The Royal Infirmary of Edinburgh
7. Alexander Bell, Sheffield Teaching Hospitals NHS Foundation Trust
8. Amy Sadler, NHS Tayside
9. Andrea Harvey, Aberdeen Royal Infirmary, NHS Grampian
10. Andrew Mitchell, NHS Lanarkshire
11. Andrew Tambyraja, Royal Infirmary of Edinburgh & University of Edinburgh
12. Andrew William Garnham, The Royal Wolverhampton NHS Trust
13. Andrey Varvinskiy, Torbay and South Devon NHS Healthcare Foundation Trust
14. Ankur Thapar, Anglia Ruskin University
15. Anna Celnik, Aberdeen Royal Infirmary, NHS Grampian
16. Caroline Curry, Royal Victoria Hospital, Belfast
17. Catherine Western, Derriford Hospital, Plymouth
18. Catriona Semple, NHS Fife and Tayside
19. Christopher G Davies, Swansea Bay University Health board
20. Craig Forrest, NHS Greater Glasgow and Clyde
21. David Bosanquet, South East Wales Vascular Network & Cardiff University
22. Denis Harkin, Belfast Health & Social Care Trust
23. Diane Rosemary Hildebrand, Cambridge University Hospitals
24. Douglas James MacKenzie, Ninewells Hospital and Medical School, Dundee
25. Emma Elizabeth Florence Scott, Sunderland Royal Hospital
26. Emma Jannine Baird, Lancashire Teaching Hospitals NHS Foundation Trust
27. Ferdinand Serracino-Inglott, Manchester Academic Health Science Centre, Manchester Royal Infirmary Manchester Vascular Centre
28. Fiona Myint, Royal Free Hospital
29. Frances Kent, East of Scotland Vascular Network
30. Francesco Torella, Liverpool Vascular & Endovascular Service, UK
31. Gareth Harrison, Countess of Chester Hospital
32. George A. Antoniou, Manchester University NHS Foundation Trust
33. George Edward Smith, Hull York Medical School
34. Graeme A McLeod, NHS Tayside
35. Ian Chetter, Hull York Medical School
36. Indran Raju, Queen Elizabeth University Hospital, NHS Greater Glasgow and Clyde
37. John Chalmers, Aberdeen Royal Infirmary, NHS Grampian
38. John Nagy, Department of Vascular Surgery, Ninewells Hospital, NHS Tayside
39. John O'Donoghue, University Hospital Hairmyres
40. Jonathan Paul Seeley, University of Dundee
41. Kaji Sritharan, Department of Surgery & Cancer, Faculty of Medicine, Imperial College London
42. Keith Hussey, Queen Elizabeth University Hospital, NHS Greater Glasgow and Clyde
43. Keith Jones, Frimley Health NHS Foundation Trust
44. Kersten Morgan Bates, East and North Hertfordshire NHS Trust
45. Kevin Graham Mercer, Bradford Teaching Hospitals NHS Foundation Trust
46. Kieran Murphy, Sheffield Teaching Hospitals NHS Foundation Trust
47. Lisa Anne Grimes, Cambridge University Hospitals NHS Foundation Trust
48. Matt Metcalfe, East and North Herts NHS Trust
49. Maureen Sweeney, NHS Lanarkshire & University of Glasgow
50. Michael McCusker, NHS Lanarkshire
51. Michael Neil, Ninewells Hospital and Medical School
52. Michelle Lamont, Aberdeen Royal Infirmary, NHS Grampian
53. Mohamed Elsherif, Sheffield Teaching Hospitals NHS Foundation Trust
54. Nat Haslam, South Tyneside and Sunderland NHS Foundation Trust
55. Naveeta Maini, NHS Tayside
56. Nicholas Stuart Greaves, Manchester Royal Infirmary, Manchester University NHS Foundation Trust
57. Patrick Coughlin, Leeds Teaching Hospital NHS Trust
58. Patrice Forget, Epidemiology group, University of Aberdeen & Aberdeen Royal Infirmary, NHS Grampian
59. Peter Merjavy, Anaesthetic Department, Southern Health and Social Care Trust, Portadown
60. Robert Hinchliffe, University of Bristol
61. Rob Sayers, Glenfield Hospital, Leicester
62. Ross Thomson, Aberdeen Royal Infirmary, NHS Grampian
63. Russell William Jamieson, Edinburgh Vascular Surgical Services, Royal Infirmary of Edinburgh
64. Sachin R Kulkarni, Gloucestershire Hospitals NHS Foundation Trust
65. Sandeep Bahia, Kent and Canterbury Hospital
66. Sandip Nandhra, Newcastle University
67. Serena Goon, Cambridge University Hospitals NHS Foundation Trust
68. Tanim Siddiqui, NHS Lanarkshire
69. Tim Stansfield, Leeds Teaching Hospitals NHS Trust
70. Vanessa Fludder, University Hospitals Sussex
71. Vikas Kaura, Leeds Institute of Medical Research, University of Leeds
72. Vishal Gupta, NHS Greater Glasgow and Clyde
