# Supplementary material for: Peri‐operative pain management in major lower extremity amputation in vascular Surgery: a UK anaesthetic and vascular surgery Delphi consensus study*
Source: Anaesthesia. 2025 Dec 17;81(5):664–74. doi: 10.1111/anae.70107 (PMC13065886; doi:10.1111/anae.70107)
Supplement: Supplementary file 2 — Appendix S2. Delphi rounds 1–3 results breakdown. Appendix S3. Thematic summary of panellist free text comments. [file ANAE-81-664-s001.docx]

**Appendix S2:** Delphi rounds 1–3 results breakdown

**Round 1 results**

|  | Statements | 1 | 2 | 3 | 4 | 5 | 4 + 5 | n= |
| --- | --- | --- | --- | --- | --- | --- | --- | --- |
|  | **1 General principles, processes, and approaches** |  |  |  |  |  |  |  |
| 1.1 | Elucidating the best practices for pharmacologically managing pain in dysvascular major lower extremity amputation patients (i.e. pre-, peri-, and post-amputation pain, including acute and chronic phantom limb pain) remains to be a key clinical practice challenge and research priority. | 0.0% | 1.4% | 1.4% | 37.5% | 59.7% | 97.2% | 72 |
| 1.2 | Existing international, national, or local guidance relating to multimodal analgesic practices are not adequately comprehensive or specific to major lower extremity amputation pain management. | 0.0% | 5.6% | 15.3% | 54.2% | 25.0% | 79.2% | 72 |
| 1.3 | Multimodal pain management practices in major lower extremity amputation vary and are not fully guided by direct evidence. | 1.4% | 2.8% | 5.6% | 59.7% | 30.6% | 90.3% | 72 |
| 1.4 | More qualitative research on patient experiences and attitudes towards multimodal perioperative amputation pain management will help inform on analgesic strategies. | 0.0% | 0.0% | 6.9% | 36.1% | 56.9% | 93.1% | 72 |
| 1.5 | Perioperative multimodal analgesic management in major lower extremity amputation is a shared responsibility between the anaesthetics, pain, and surgical teams. | 0.0% | 0.0% | 0.0% | 18.1% | 81.9% | 100.0% | 72 |
| 1.6 | Perioperative pain management strategies in major lower extremity amputation patients should be guided by pain specialists. | 4.2% | 13.9% | 25.0% | 44.4% | 12.5% | 56.9% | 72 |
| 1.7 | The approaches to major lower extremity amputation pain management should be personalised by considering biopsychosocial patient factors. | 0.0% | 2.8% | 18.1% | 41.7% | 37.5% | 79.2% | 72 |
| 1.8 | Analgesic initiation and escalation in post-amputation pain should be titrated according to acute functional performance and biopsychosocial outcomes in addition to pain scores. | 0.0% | 4.2% | 12.5% | 41.7% | 41.7% | 83.3% | 72 |
| 1.9 | Pre-operative pain management in patients undergoing major lower extremity amputation affects post-amputation pain outcomes and should be as equally prioritised in routine clinical practice as post-amputation pain. | 0.0% | 0.0% | 2.8% | 31.9% | 65.3% | 97.2% | 72 |
| 1.10 | Collaborative protocols involving the pain and surgical teams should be developed to facilitate early identification of patients due for major lower extremity amputation to initiate early pre-operative analgesic review and optimisation. | 1.4% | 0.0% | 4.2% | 26.4% | 68.1% | 94.4% | 72 |
| 1.11 | Those who do not identify themselves as pain specialists but are involved in the clinical management of lower extremity amputation patients should be equipped with competencies to initiate, titrate, and recognise the complications of certain non-opioid multimodal analgesic agents. | 0.0% | 1.4% | 6.9% | 20.8% | 70.8% | 91.7% | 72 |
| 1.12 | Decision aids for the perioperative management of pain should be made accessible for non-pain specialists. | 0.0% | 0.0% | 5.6% | 12.5% | 81.9% | 94.4% | 72 |
| 1.13 | Perioperative pain management pathways for major lower extremity amputation patients should be adapted locally to multispecialty departmental consensus and experience. | 0.0% | 2.8% | 6.9% | 38.9% | 51.4% | 90.3% | 72 |
| 1.14 | Patient education on post-amputation pain symptoms, analgesic strategies, and side effects should be incorporated into routine patient contact. | 0.0% | 1.4% | 2.8% | 22.2% | 73.6% | 95.8% | 72 |
| 1.15 | Where pain services are subdivided, acute pain specialists involved with post-amputation pain management should be equipped with chronic pain management competencies for continuity. | 1.4% | 4.2% | 15.3% | 36.1% | 43.1% | 79.2% | 72 |
|  | **2 Locoregional and intraoperative approaches** |  |  |  |  |  |  |  |
| 2.1 | Locoregional analgesia should be the mainstay modality of pain management, where available, in major lower extremity amputations pre-, intra-, and post-operatively. | 0.0% | 1.5% | 10.4% | 26.9% | 61.2% | 88.1% | 67 |
| 2.2 | Continuous local anaesthetic infusions via a perineural nerve catheter is the preferred locoregional analgesia modality over single nerve blocks. | 0.0% | 0.0% | 10.4% | 26.9% | 62.7% | 89.6% | 67 |
| 2.3 | Perineural catheter placement under ultrasound guidance may provide greater analgesic reliability than intraoperative placement under direct visualisation, where expertise is available. | 7.8% | 28.1% | 34.4% | 12.5% | 17.2% | 29.7% | 64 |
| 2.4 | Percutaneous perineural catheter placement under ultrasound guidance should be particularly indicated where there are concerns with amputation site infection. | 10.8% | 9.2% | 16.9% | 30.8% | 32.3% | 63.1% | 65 |
| 2.5 | Continuous local anaesthetic infusion by perineural nerve catheters should be initiated pre-operatively. | 4.5% | 18.2% | 24.2% | 25.8% | 27.3% | 53.0% | 66 |
| 2.6 | Continuous local anaesthetic infusion by perineural nerve catheters should be continued for an extended acute post-operative duration (e.g. more than 72 hours). | 6.1% | 1.5% | 15.2% | 19.7% | 57.6% | 77.3% | 66 |
| 2.7 | In above-knee amputations, continuous local anaesthetic infusions should routinely target both the femoral and sciatic nerves. | 1.6% | 20.3% | 12.5% | 20.3% | 45.3% | 65.6% | 64 |
| 2.8 | In below-knee amputations, the perineural nerve catheter should target the sciatic nerve proximally via ultrasound guidance, at or above the popliteal fossa. | 3.1% | 14.1% | 18.8% | 17.2% | 46.9% | 64.1% | 64 |
| 2.9 | In below-knee amputations, continuous perineural tibial nerve catheters placed intraoperatively under direct vision may not provide complete anatomical coverage but may be preferable for practical reasons. | 1.6% | 4.7% | 17.2% | 48.4% | 28.1% | 76.6% | 64 |
| 2.10 | Where there may be contraindications to perineural nerve catheter placement, neuraxial (e.g. epidural) or alternative peripheral locoregional techniques such as single nerve blocks should be routinely employed. | 0.0% | 7.7% | 6.2% | 27.7% | 58.5% | 86.2% | 65 |
| 2.11 | Intraoperative nerve transection techniques in tractional neurectomy vary by preference, but the body of evidence to support their effect on acute and chronic pain outcomes are scarce and equivocal. | 0.0% | 2.4% | 14.6% | 41.5% | 41.5% | 82.9% | 41 |
| 2.12 | The benefit of intraoperatively incorporating alternative nerve-handling techniques: targeted muscle reinnervation, regenerative peripheral nerve interface, or targeted nerve implantation, on pain outcomes should be investigated for feasibility and implementability. | 0.0% | 8.7% | 17.4% | 39.1% | 34.8% | 73.9% | 46 |
|  | **3 Pharmacological approaches** |  |  |  |  |  |  |  |
| 3.1 | The co-morbidities and potential risks of polypharmacy in dysvascular patients undergoing major lower extremity amputation are barriers to the initiation, titration, and escalation of multimodal analgesic agents. | 3.2% | 7.9% | 11.1% | 58.7% | 19.0% | 77.8% | 63 |
| 3.2 | Multimodal analgesia in patients undergoing major lower extremity amputation should aim to be opioid-sparing as much as possible. | 0.0% | 3.1% | 9.4% | 51.6% | 35.9% | 87.5% | 64 |
| 3.3 | The choice of pharmacological analgesic agents in major lower extremity amputation pain management should be personalised by considering biopsychosocial patient factors. | 0.0% | 1.6% | 3.2% | 41.3% | 54.0% | 95.2% | 63 |
| 3.4 | Opioids should be incorporated as a rescue medication and should not be prescribed as continuous infusion or using slow-release formulation as the mainstay of pharmacological post-amputation and phantom limb pain management. | 1.6% | 11.1% | 22.2% | 27.0% | 38.1% | 65.1% | 63 |
| 3.5 | The potential adverse effects arising from utilising non-opioid multimodal analgesic agents are a greater concern to me than the adverse effects arising from opioids. | 12.7% | 28.6% | 34.9% | 22.2% | 1.6% | 23.8% | 63 |
| 3.6 | The choice of pharmacological analgesic strategy I employ or prefer depends on their potential impact on chronic and phantom limb pain outcomes. | 1.6% | 6.5% | 14.5% | 58.1% | 19.4% | 77.4% | 62 |
| 3.7 | NSAIDs are efficacious and could be (appropriately) used more often in the management of acute nociceptive post-amputation pain. | 1.6% | 20.6% | 14.3% | 30.2% | 33.3% | 63.5% | 63 |
| 3.8 | Nefopam should always (if not contraindicated) be employed as an adjunctive analgesic agent for acute post-amputation pain. | 22.5% | 22.5% | 30.0% | 22.5% | 2.5% | 25.0% | 40 |
| 3.9 | Pre-operative initiation of (pre-medication with) gabapentinoids should be routine unless there are concerns with increased sedation risk. | 8.3% | 8.3% | 18.3% | 30.0% | 35.0% | 65.0% | 60 |
| 3.10 | Oral alpha-2 agonists (e.g. clonidine) should be routinely incorporated into the analgesic management regime as an escalation and rescue measure, particularly in non-opioid naïve patients, opioid and opiate withdrawal, or in patients with anxiety. | 4.5% | 9.1% | 20.5% | 45.5% | 20.5% | 65.9% | 44 |
| 3.11 | Oral NMDA receptor antagonists (e.g. ketamine) should be routinely incorporated into the multimodal analgesic management regime as an escalation and rescue measure, particularly where there are high risks of opioid tolerance or phantom limb pain. | 8.9% | 4.4% | 24.4% | 35.6% | 26.7% | 62.2% | 45 |
| 3.12 | Parenteral calcitonin has a favourable safety profile and should be incorporated as an acute post-amputation pain management measure. | 7.7% | 26.9% | 34.6% | 15.4% | 15.4% | 30.8% | 26 |
| 3.13 | De-escalation and weaning regimes for perineural catheters and all non-opioid analgesics should be specific, standardised, and readily accessible. | 0.0% | 1.6% | 4.7% | 26.6% | 67.2% | 93.8% | 64 |

**Round 2 results**, statement numbers highlighted in blue denote statements that were revised or newly introduced.

|  | Statements | 1 | 2 | 3 | 4 | 5 | 4 + 5 | n= |
| --- | --- | --- | --- | --- | --- | --- | --- | --- |
|  | **1 General principles, processes, and approaches** |  |  |  |  |  |  |  |
| 1.1 | Elucidating the best practices for pharmacologically managing pain in dysvascular major lower extremity amputation patients (i.e. pre-, peri-, and post-amputation pain, including acute and chronic phantom limb pain) remains to be a key clinical practice challenge and research priority. | 0.0% | 0.0% | 1.5% | 32.8% | 65.7% | 98.5% | 67 |
| 1.2 | Existing international, national, or local guidance relating to multimodal analgesic practices are not adequately comprehensive or specific to major lower extremity amputation pain management. | 0.0% | 3.0% | 11.9% | 62.7% | 22.4% | 85.1% | 67 |
| 1.3 | Multimodal pain management practices in major lower extremity amputation vary and are not fully guided by direct evidence. | 0.0% | 1.5% | 6.0% | 67.2% | 25.4% | 92.5% | 67 |
| 1.4 | More qualitative research on patient experiences and attitudes towards multimodal perioperative amputation pain management will help inform on analgesic strategies. | 0.0% | 0.0% | 4.5% | 31.3% | 64.2% | 95.5% | 67 |
| 1.5 | Perioperative multimodal analgesic management in major lower extremity amputation is a shared responsibility between the anaesthetics, pain, and surgical teams. | 0.0% | 0.0% | 1.5% | 7.5% | 91.0% | 98.5% | 67 |
| **1.6** | Perioperative pain management strategies in major lower extremity amputation patients should be guided by pain specialists. | 0.0% | 9.0% | 26.9% | 50.7% | 13.4% | 64.2% | 67 |
| **1.7** | The approaches to major lower extremity amputation pain management should be personalised by considering biopsychosocial patient factors. | 0.0% | 0.0% | 10.4% | 47.8% | 41.8% | 89.6% | 67 |
| 1.8 | Analgesic initiation and escalation in post-amputation pain should be titrated according to acute functional performance and biopsychosocial outcomes in addition to pain scores. | 0.0% | 0.0% | 7.5% | 46.3% | 46.3% | 92.5% | 67 |
| 1.9 | Pre-operative pain management in patients undergoing major lower extremity amputation affects post-amputation pain outcomes and should be as equally prioritised in routine clinical practice as post-amputation pain. | 0.0% | 0.0% | 0.0% | 17.9% | 82.1% | 100.0% | 67 |
| 1.10 | Collaborative protocols involving the pain and surgical teams should be developed to facilitate early identification of patients due for major lower extremity amputation to initiate early pre-operative analgesic review and optimisation. | 0.0% | 0.0% | 1.5% | 7.5% | 91.0% | 98.5% | 67 |
| 1.11 | Those who do not identify themselves as pain specialists but are involved in the clinical management of lower extremity amputation patients should be equipped with competencies to initiate, titrate, and recognise the complications of certain non-opioid multimodal analgesic agents. | 0.0% | 1.5% | 1.5% | 11.9% | 85.1% | 97.0% | 67 |
| 1.12 | Decision aids for the perioperative management of pain should be made accessible for non-pain specialists. | 0.0% | 0.0% | 4.5% | 14.9% | 80.6% | 95.5% | 67 |
| 1.13 | Perioperative pain management pathways for major lower extremity amputation patients should be adapted locally to multispecialty departmental consensus and experience. | 0.0% | 3.0% | 1.5% | 29.9% | 65.7% | 95.5% | 67 |
| 1.14 | Patient education on post-amputation pain symptoms, analgesic strategies, and side effects should be incorporated into routine patient contact. | 0.0% | 0.0% | 1.5% | 13.4% | 85.1% | 98.5% | 67 |
| **1.15** | Where pain services are subdivided, acute pain specialists involved with post-amputation pain management should be equipped with chronic pain management competencies for continuity. | 1.5% | 1.5% | 10.4% | 40.3% | 46.3% | 86.6% | 67 |
|  | **2 Locoregional and intraoperative approaches** |  |  |  |  |  |  |  |
| 2.1 | Locoregional analgesia should be the mainstay modality of pain management, where available, in major lower extremity amputations pre-, intra-, and post-operatively. | 0.0% | 0.0% | 3.2% | 29.0% | 67.7% | 96.8% | 62 |
| 2.2 | Continuous local anaesthetic infusions via a perineural nerve catheter is the preferred locoregional analgesia modality over single nerve blocks. | 0.0% | 0.0% | 10.0% | 13.3% | 76.7% | 90.0% | 60 |
| **2.3** | Perineural catheter placement under ultrasound guidance may provide greater analgesic reliability than intraoperative placement under direct visualisation, where expertise is available. | 5.1% | 25.4% | 40.7% | 15.3% | 13.6% | 28.8% | 59 |
| **2.4** | Percutaneous perineural catheter placement under ultrasound guidance should be particularly indicated where there are concerns with amputation site infection. | 8.2% | 3.3% | 21.3% | 34.4% | 32.8% | 67.2% | 61 |
| **2.5** | Continuous local anaesthetic infusion by perineural nerve catheters or other locoregional analgesia modalities should routinely be initiated when surgical indication for amputation is confirmed. | 1.6% | 16.4% | 27.9% | 29.5% | 24.6% | 54.1% | 61 |
| **2.6** | Continuous local anaesthetic infusion by perineural nerve catheters should be continued for an extended acute post-operative duration (e.g. more than 72 hours). | 4.9% | 1.6% | 9.8% | 11.5% | 72.1% | 83.6% | 61 |
| **2.7** | In above-knee amputations, continuous local anaesthetic infusions should routinely target both the femoral and sciatic nerves. | 0.0% | 13.6% | 10.2% | 23.7% | 52.5% | 76.3% | 59 |
| 2.8 | In below-knee amputations, the perineural nerve catheter should target the sciatic nerve proximally via ultrasound guidance, at or above the popliteal fossa. | 0.0% | 11.7% | 13.3% | 16.7% | 58.3% | 75.0% | 60 |
| 2.9 | In below-knee amputations, continuous perineural tibial nerve catheters placed intraoperatively under direct vision may not provide complete anatomical coverage but may be preferable for practical reasons. | 0.0% | 5.1% | 13.6% | 52.5% | 28.8% | 81.4% | 59 |
| 2.10 | Where there may be contraindications to perineural nerve catheter placement, neuraxial (e.g. epidural) or alternative peripheral locoregional techniques such as single nerve blocks should be routinely employed. | 0.0% | 0.0% | 6.6% | 16.4% | 77.0% | 93.4% | 61 |
| 2.11 | Intraoperative nerve transection techniques in tractional neurectomy vary by preference, but the body of evidence to support their effect on acute and chronic pain outcomes are scarce and equivocal. | 0.0% | 0.0% | 10.3% | 33.3% | 56.4% | 89.7% | 39 |
| 2.12 | The benefit of intraoperatively incorporating alternative nerve-handling techniques: targeted muscle reinnervation, regenerative peripheral nerve interface, or targeted nerve implantation, on pain outcomes should be investigated for feasibility and implementability. | 0.0% | 4.7% | 16.3% | 39.5% | 39.5% | 79.1% | 43 |
| 2.13 | Epidural analgesia should be considered as a locoregional analgesic modality in the ward setting pre- and post-operatively. | 8.1% | 30.6% | 16.1% | 30.6% | 14.5% | 45.2% | 62 |
|  | **3 Pharmacological approaches** |  |  |  |  |  |  |  |
| 3.1 | The co-morbidities and potential risks of polypharmacy in dysvascular patients undergoing major lower extremity amputation are barriers to the initiation, titration, and escalation of multimodal analgesic agents. | 1.7% | 1.7% | 11.9% | 62.7% | 22.0% | 84.7% | 59 |
| 3.2 | Multimodal analgesia in patients undergoing major lower extremity amputation should aim to be opioid-sparing as much as possible. | 0.0% | 0.0% | 6.8% | 59.3% | 33.9% | 93.2% | 59 |
| 3.3 | The choice of pharmacological analgesic agents in major lower extremity amputation pain management should be personalised by considering biopsychosocial patient factors. | 0.0% | 0.0% | 1.7% | 37.9% | 60.3% | 98.3% | 58 |
| 3.4 | Immediate release opioids are recommended as a rescue medication measure. Continuous infusion or slow-release formulations are not recommended as the mainstay of post-amputation and phantom limb pain management. | 0.0% | 5.2% | 22.4% | 27.6% | 44.8% | 72.4% | 58 |
| **3.5** | The potential adverse effects arising from utilising non-opioid multimodal analgesic agents are a greater concern to me than the adverse effects arising from opioids. | 11.9% | 30.5% | 35.6% | 20.3% | 1.7% | 22.0% | 59 |
| 3.6 | The choice of pharmacological analgesic strategy I employ or prefer depends on their potential impact on chronic and phantom limb pain outcomes. | 0.0% | 3.5% | 10.5% | 71.9% | 14.0% | 86.0% | 57 |
| **3.7** | NSAIDs are efficacious and could be appropriately (meaning respecting the frequent absolute and relative contraindications) used more often in the management of acute nociceptive post-amputation pain. | 1.7% | 15.3% | 8.5% | 37.3% | 37.3% | 74.6% | 59 |
| **3.8** | Nefopam should always (if not contraindicated) be employed as an adjunctive analgesic agent for acute post-amputation pain. | 26.3% | 13.2% | 39.5% | 21.1% | 0.0% | 21.1% | 38 |
| **3.9** | Pre-operative initiation of (pre-medication with) gabapentinoids should be routine unless there are concerns with increased sedation risk. | 9.1% | 3.6% | 23.6% | 29.1% | 34.5% | 63.6% | 55 |
| **3.10** | Oral alpha-2 agonists (e.g. clonidine) should be routinely incorporated into the analgesic management regime as an escalation and rescue measure, particularly in non-opioid naïve patients, opioid and opiate withdrawal, or in patients with anxiety. | 4.9% | 7.3% | 22.0% | 51.2% | 14.6% | 65.9% | 41 |
| **3.11** | Oral NMDA receptor antagonists (e.g. low dose oral ketamine) should be routinely incorporated into the multimodal analgesic management regime as an escalation and rescue measure, particularly where there are high risks of opioid tolerance or phantom limb pain. | 7.1% | 2.4% | 26.2% | 40.5% | 23.8% | 64.3% | 42 |
| **3.12** | Parenteral calcitonin has a favourable safety profile and should be incorporated as an acute post-amputation pain management measure. | 12.0% | 24.0% | 40.0% | 8.0% | 16.0% | 24.0% | 25 |
| 3.13 | De-escalation and weaning regimes for perineural catheters and all non-opioid analgesics should be specific, standardised, and readily accessible. | 0.0% | 1.7% | 5.1% | 13.6% | 79.7% | 93.2% | 59 |

**Round 3 results**, statement numbers highlighted in blue denote statements that were revised or newly introduced.

|  | Statements | 1 | 2 | 3 | 4 | 5 | 4 + 5 | n= |
| --- | --- | --- | --- | --- | --- | --- | --- | --- |
|  | **1 General principles, processes, and approaches** |  |  |  |  |  |  |  |
| **1.16** | The implementation of any potential perioperative pain management processes, approaches, or modalities in major lower extremity amputation requires an appropriate dedication of resources at the local and regional levels. | 0.0% | 2.9% | 2.9% | 23.2% | 71.0% | 94.2% | 69 |
| **1.17** | Where resources may be limited, the surgical team (i.e. surgical Registrars and Consultants) should also be equipped with competencies to deliver relevant locoregional analgesia (e.g. perineural catheters or nerve blocks). | 4.3% | 14.5% | 11.6% | 36.2% | 33.3% | 69.6% | 69 |
|  | **2 Locoregional and intraoperative approaches** |  |  |  |  |  |  |  |
| **2.7** | In above-knee amputations, continuous local anaesthetic infusions should routinely target both the femoral and sciatic nerves. | 0.0% | 8.3% | 8.3% | 26.7% | 56.7% | 83.3% | 60 |
| 2.8 | In below-knee amputations, the perineural nerve catheter should target the sciatic nerve proximally via ultrasound guidance, at or above the popliteal fossa. | 0.0% | 13.1% | 11.5% | 14.8% | 60.7% | 75.4% | 61 |
| 2.12 | The benefit of intraoperatively incorporating alternative nerve-handling techniques: targeted muscle reinnervation, regenerative peripheral nerve interface, or targeted nerve implantation, on pain outcomes should be investigated for feasibility and implementability. | 2.4% | 2.4% | 11.9% | 47.6% | 35.7% | 83.3% | 42 |
| 2.13 | Epidural analgesia should be considered as a locoregional analgesic modality in the ward setting pre- and post-operatively. | 6.3% | 40.6% | 17.2% | 26.6% | 9.4% | 35.9% | 64 |
|  | **3 Pharmacological approaches** |  |  |  |  |  |  |  |
| **3.4.1** | Immediate release opioids are recommended as a rescue medication measure in post-amputation and phantom limb pain management. | 0.0% | 0.0% | 10.2% | 30.5% | 59.3% | 89.8% | 59 |
| **3.4.2** | Continuous infusion or slow-release opioids are not recommended as the mainstay of post-amputation and phantom limb pain management. | 0.0% | 1.7% | 18.6% | 23.7% | 55.9% | 79.7% | 59 |
| **3.7** | NSAIDs are efficacious and could be appropriately (meaning respecting the frequent absolute and relative contraindications) used more often in the management of acute nociceptive post-amputation pain. | 3.3% | 6.7% | 13.3% | 35.0% | 41.7% | 76.7% | 60 |

**Appendix S3:** Thematic summary of panellist free text comments

| **Theme** | **Total comments (n)** | **Comments by round (1st/2nd/3rd)** | **Summary** | **Examples** |
| --- | --- | --- | --- | --- |
| Pre-operative analgesia | 10 | 9/1/0 | Comments recognised the importance of this theme as a priority area for consensus and research and advocate for a selective, stratified approach to preoperative analgesic optimisation. | *“Improved recognition of pre operative pain is important.”  “The value of pre-operative strategies seems like a major target for consensus on current evidence and areas for future research”*  *“I don’t think a 'blanket rule for all' is as helpful as risk stratification of likelihood for post operative pain. In other words, dysvascular major lower extremity amputation might nor in itself justify pre-op MDT input/complex post op pain management etc (over other surgeries) per se .. but if there are risk factors for post op pain than this would be helpful.”* |
|  |  |  |  |  |
| Overall analgesic approaches | 7 | 5/2/0 | Comments emphasised the difficulties in establishing a uniform analgesic approach and a need for personalisation strategies with acknowledgement of a need to address biopsychosocial factors. | *“A It would be useful to elucidate the value of techniques in differing clinical scenarios and phases of the procedure”  “All patient management decisions regarding analgesic techniques should be made on a case by case basis as there are so many variables”  “If we're talking about biopsychosocial factors, should this also be part of the question? ie earlyu psychology/OT input?”* |
| Epidurals | 6 | 0/6/0 | Comments mainly expressed concerns regarding the safety and feasibility of epidural analgesia in the routine pre and postoperative settings. | *“Some hospitals nowadays have many wards that are unable to manage epidurals and thus statement 13 would be hard to deliver in those hospitals.”  “i feel epidural pre op doesn't have enough evidence and would be very difficult to achieve in the NHS setting. plus gives you less days post op”* |
| Resources | 5 | 0/1/4 | Comments showed strong acknowledgement of resources as a potential barrier to delivering any ideal pain management strategies. | *“The implementation of any potential perioperative pain management processes, approaches, or modalities in major lower extremity amputation needs to be supported by appropriate research / evidence base prior to implementation, and if it doesn't exist this needs addressing”*  *“The latter half of the questions are very resource and finance dependant, I don't know whether this warrants a mention given the lack of these which exist in the NHS?”* |
| Role of the non-pain specialist | 4 | 4/0/0 | Contrasting views on a more supportive role of pain teams but also some concerns regarding the training and capabilities of more junior clinical team members in pain management. | *“Specialist input by acute and chronic pain teams should be an adjunct, rather than a replacement, to routine care by AHPs, non-pain anaesthetists, and vascular doctors so that adjustment of treatment can be responsive to the patient's needs.”  “Even with robust guidance in place. Routine use of adjuncts for managing escalating post-operative pain should be the domain of the acute pain team, due to the frequent rotation of junior doctors within the home surgical teams. This rotation creates issues regarding awareness of guidelines and effectiveness of treatment”* |
| Delivery of locoregional analgesia | 5 | 2/1/2 | Contrasting views on surgeon-placed perineural catheters, particularly regarding transferability of skills, training, quality control, and need for comparisons. | *“Surgeons Should be trained to place nerve catheters and similar. We have transferable ultrasound skills from vein and artery access.”  “Expecting surgeons to perform RA as described in 17 is a step too far. It's not addressing the issue of the lack of resources. Also, the RA technique is part of the bigger APS service. The suggestion introduces too much variation into the process and how do we maintain quality control?”  “Q3 can easily be moulded into a comparative study between two different modalities looking into post-operative opioid use (anaesthetic v surgical placement of catchers)”* |
| Ketamine | 3 | 2/1/0 | Comments mainly expressed concerns regarding the use of parenteral Ketamine. | *“Patients usually have ward care post op so ketamine isn't really an option.”*  *“ketamine - oral verse low dose infusion (s/c or iv)”* |
| Nerve handling | 3 | 2/0/1 | Comments expressed uncertainty about optimal nerve handling techniques intraoperatively and a need for more evidence. | *“Evidence on how to leave the nerve would be beneficial.”* |
| Opioids | 3 | 1/2/0 | Concerns about opioid escalation and subsequent weaning and a need to address immediate and prolonged opioids separately. | *“One of the most challenging aspects I find is the rapid escalation of opioids several days post-operatively, particularly in a vulnerable population (many have substance misuse issues) and the subsequent weaning of these drugs.”* |
| Gabapentinoids | 3 | 2/1/0 | Comments demonstrated reservations regarding efficacy and appropriateness of routine use due to a lack of definitive evidence. | *“I've not used pre-med gabapentinoids routinely but would be particularly interested in whether this is valuable in all or selected cases”* |
| NSAIDs | 2 | 2/0/0 | Comments demonstrated emphasis on risk-benefit balance of use in vascular surgical patients with openness to further population-specific evidence. | *“We tend to avoid NSAIDs due to high risk of peptic ulceration and CKD in lots of patients. I would be happy to participate in and be educated by a randomised trial in amputation patients.”* |
